# TEL2 suppresses metastasis by down-regulating SERPINE1 in nasopharyngeal carcinoma

**DOI:** 10.18632/oncotarget.5074

**Published:** 2015-08-13

**Authors:** Yi Sang, Ming-yuan Chen, Donghua Luo, Ru-Hua Zhang, Li Wang, Mei Li, Rongzhen Luo, Chao-Nan Qian, Jian-Yong Shao, Yi-Xin Zeng, Tiebang Kang

**Affiliations:** ^1^ State Key Laboratory of Oncology in South China, Collaborative Innovation Center for Cancer Medicine, Sun Yat-sen University Cancer Center, Guangzhou, China; ^2^ Nanchang Key Laboratory of Cancer Pathogenesis and Translational Research, The Third Affiliated Hospital, Nanchang University, Nanchang, China

**Keywords:** nasopharyngeal carcinoma, metastasis, TEL2, SERPINE1

## Abstract

Metastasis is the major cause of treatment failure in patients with nasopharyngeal carcinoma (NPC). However, the molecular mechanisms of NPC metastasis are poorly understood. Here, using our customized gene microarray containing all of the known human transcription factors and the current markers for epithelial-mesenchymal transition, we report that TEL2 was down-regulated in highly metastatic NPC cells and the metastatic tissues in lymph node. Mechanistically, TEL2 inhibits the cell migration and invasion *in vitro* and metastasis *in vivo* by releasing its direct suppression on the SERPINE1 promoter in NPC. Consistently, an inverse correlation was observed between the protein levels of TEL2 and SERPINE1 using clinical NPC samples. Collectively, we have provided the first evidence that TEL2 plays a key role in NPC metastasis by directly down-regulating SERPINE1, and that this novel axis of TEL2 / SERPINE1 may be valuable to develop new strategies for treating NPC patients with metastasis.

## INTRODUCTION

Nasopharyngeal carcinoma (NPC), originating from the nasopharynx, is highly prevalent in Southern China and Southeast Asia with an incidence rate of 15–50/100,000 [1–4]. NPC has the highest metastasis rate among head and neck cancers, with 74.5% and 19.9% of patients presenting with regional lymph node metastasis and distant metastasis including liver and lung at the time of diagnosis, respectively. Distant metastasis is the major cause of treatment failure for NPC patients [1, 3, 5, 6]. Furthermore, the rate of 5-year accumulated distant metastasis was much greater in the T1-T2N1 group than that in the T1-T2N0 group, according to the 1992 Chinese staging system for patients with NPC, with the result that the 5-year survival rate is much lower in the former group [7]. Although some progress has been made recently, the molecular mechanisms of NPC metastasis are poorly understood [8, 9].

Many transcription factors play a key role in regulating cancer metastasis. For instance, cancer metastasis is [10, 11] inhibited by p53 but promoted by ZEB1. To determine the roles of these factors in NPC metastasis, we have generated the customized gene microarray containing all of the known human transcription factors and the current markers for epithelial-mesenchymal transition (EMT). Using this special microarray, TEL2, an ETS family whose members have key roles in cancer metastasis [12–15], was identified as an important regulator of NPC metastasis.

TEL2 is one of the ETS (E26-transformation specific) families of transcription factors. This family has highly similar DNA-binding ETS domains that recognize a purine-rich GGAA core motif within the promoters and enhancers of various target genes, and positively and/or negatively regulates the expression of target genes that have diverse functions and activities in signaling pathways, development, cell proliferation, differentiation, migration, apoptosis, invasion and metastasis [12, 13]. Among the ETS proteins, TEL2 is highly homologous to TEL in both sequence and structure, with both proteins containing an amino-(N-) terminal PNT domain that is thought to mediate protein-protein interactions, and they are the transcriptional repressors [16–19]. Most of the work on TEL2 have been focused on normal hematopoiesis and leukemia, and there is no evidence in the literature documenting its role in cancer metastasis [16, 17, 19]. In this study, we demonstrate that TEL2 plays a key role in NPC metastasis by directly down-regulating SERPINE1, and that this novel axis of TEL2 / SERPINE1 may be valuable to develop new strategies for treating NPC patients with metastasis.

## RESULTS

### TEL2 is down-regulated in the metastatic cells and tissues of NPC

**Figure 1:**
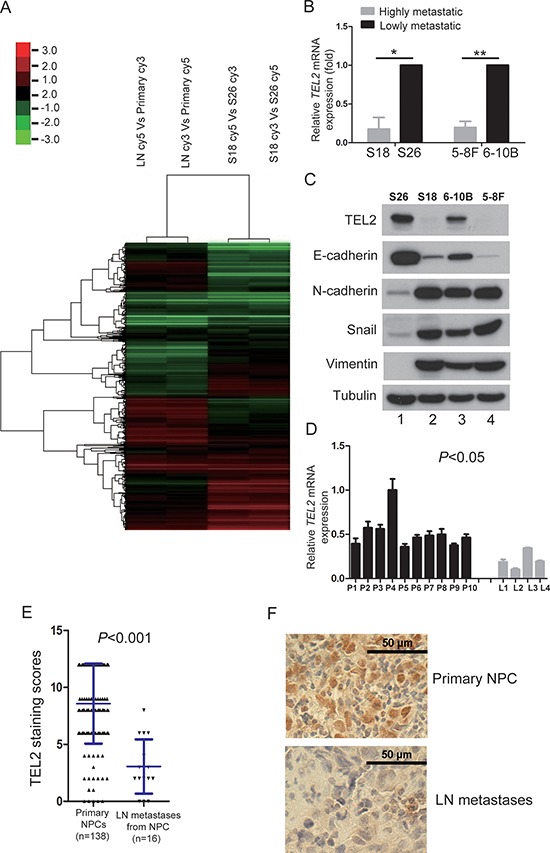
TEL2 is down-regulated in the metastatic cells and tissues of NPC **A.** The expression profiles for human transcription factors and EMT markers in the indicted cell lines using the microarray as described in “Methods”. Primary: primary tumor tissues, LN: the metastatic tumor tissues in lymph node. **B.** The relative mRNA levels of *TEL2* normalized to GAPDH level in the indicated cell lines, as determined by qRT-PCR. *n* = 3. The bars indicate the SD. *P < 0.05 and **P < 0.001 using Student's *t*-test. **C.** The proteins in the indicated cell lines were analyzed by Western blotting. **D.** The mRNA levels of *TEL2* in the indicated tissues were measured as presented in b. P: NPC primary tissues, *n* = 10, L: the metastatic tumor tissues in lymph node, *n* = 4. The bars indicate the SD. Statistical analysis (Mann-Whitney test) was performed (*P* < 0.05). **E, F.** IHC for the clinical NPC samples of both primary sites and the metastatic tumor tissues in lymph node. The panel F presents representative images, while the panel E illustrates the statistical results using a Mann-Whitney test (*P* < 0.05). The dots represent the scores, while the bars indicate the SD. Primary NPC: primary NPC tissues, *n* = 138, LN metastases: the metastatic tumor tissues from NPC in lymph node, *n* = 16. Scale bars in F, 50 μm.

To explore the roles of the transcriptional factors and the known markers for EMT in NPC metastasis, we generated the special gene microarray containing these elements. Using this microarray, we performed screenings for the pair of NPC primary tumor tissues (primary) and its metastatic tissues in lymph node (LN), and for two cell lines of S18 and S26 derived from the NPC cell line CNE2 with highly and lowly metastatic abilities, respectively, [8] as shown in Figure 1A. Although many of these gene expressions were different between the primary tumor and the LN and/or those of S26 and S18, as shown in Supplemental Table 1, there were only small percentages of genes simultaneously altered in both sets of screens, including the well-known markers for EMT and/or metastasis, such as Snail, Twist and Zeb1, indicating that this strategy was suitable to look for new regulators for NPC metastasis. Interestingly, TEL2, an ETS family whose members have key role in cancer metastasis [12, 13], was revealed to be one of the most altered transcription factors.

Using S18, S26 and two other cell lines of 5–8F and 6–10B derived from the NPC Sune1 cell line with highly and lowly metastatic abilities, respectively, both mRNA and protein levels of TEL2 were found to be lower in the highly metastatic cell lines compared with the poorly metastatic cell lines (Figures 1B and 1C). Using clinical NPC samples, TEL2 was higher in NPC primary tissues compared with the metastatic LN tissues both at the mRNA level as quantified by qRT-PCR and at the protein level as quantified by immunohistochemistry (IHC) (Figures 1D–1F).

Next, we asked whether the pattern of TEL2 in different metastatic abilities of NPC cell lines and tumor tissues would be extended to other cancer types. Interestingly, using SW480 and SW620 cell lines, which were from the primary site and the metastatic LN tissue of the same patient with colorectal cancer, respectively, both mRNA and protein levels of TEL2 were higher in SW480 cells that those in SW620 cells (Supplementary Figures S1A and S1B). Furthermore, the TEL2 protein levels were higher in the colon cancer primary tissues than that in their paired LN metastases (Supplementary Figure S1C). Therefore, it may be a general phenomenon that TEL2 is down-regulated in metastatic cancer cell lines and tissues.

### TEL2 is a negative regulator of NPC metastasis

**Figure 2:**
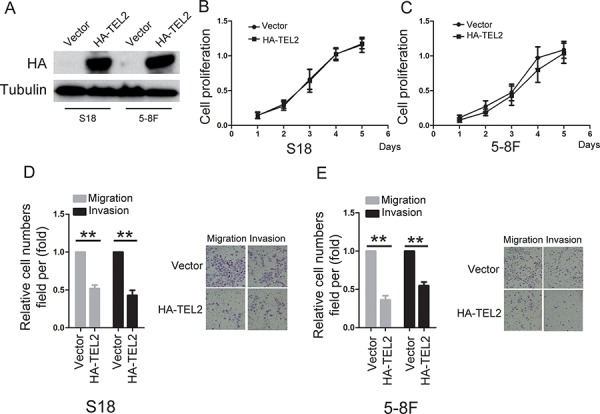
Overexpression of TEL2 suppresses migration and invasion of NPC cells **A.** The proteins were analyzed in the indicted stable cell lines. **B, C.** Cell proliferation was measured in the indicted stable cell lines by the CCK8 assay. *n* = 3. **D, E.** Cell migration and invasion were determined in the indicated stable cell lines overexpressing TEL2 as described in “Methods”. *n* = 3. The bars indicate the SD. **P < 0.001 using Student's *t*-test. The representative images (100×) for the indicated stable cell lines were shown as the left panels.

**Figure 3:**
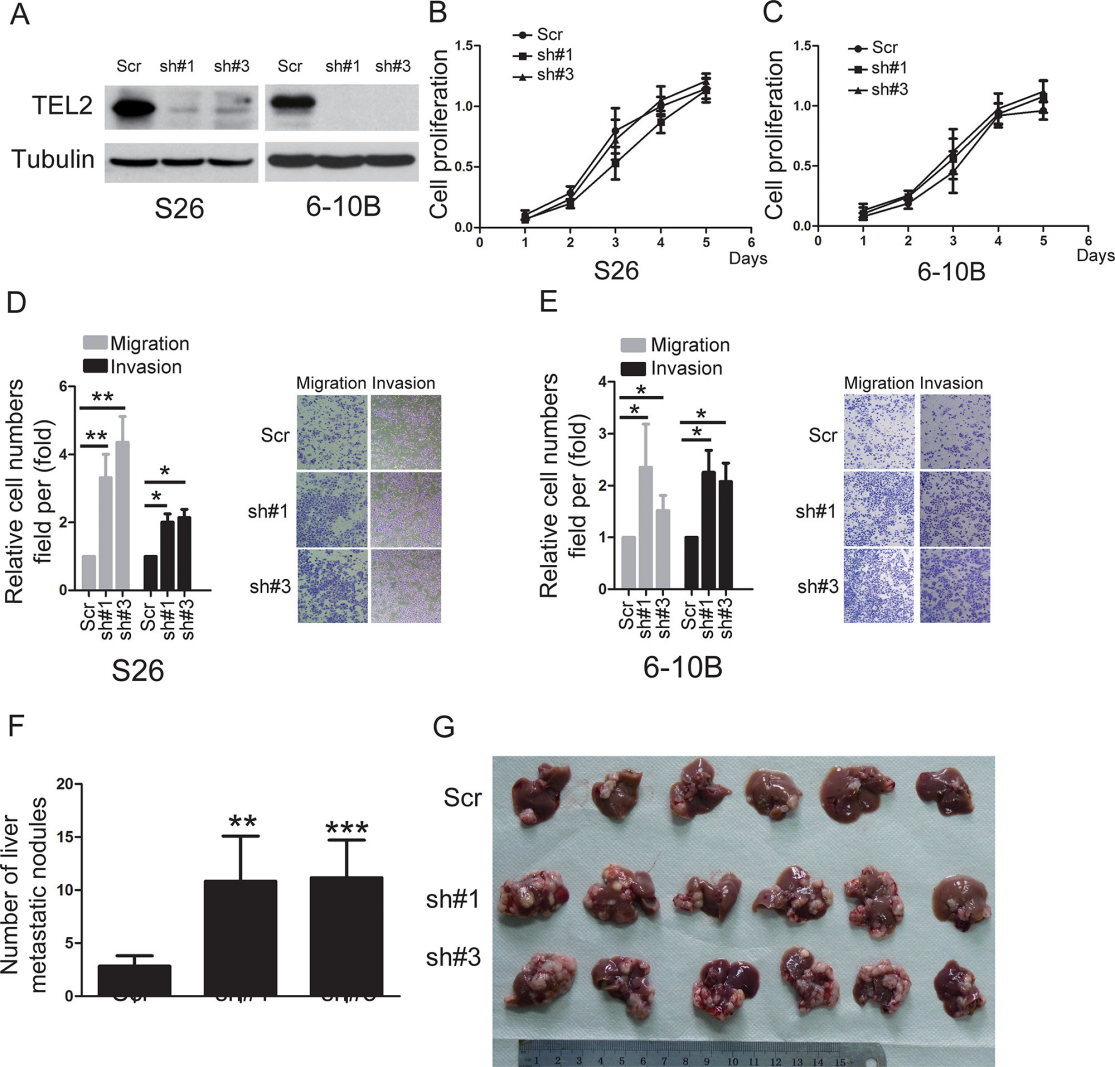
knockdown of TEL2 promotes NPC metastasis **A.** The proteins were analyzed in the indicted stable cell lines. **B, C.** Cell proliferation was measured in the indicted stable cell lines by the CCK8 assay. *n* = 3. **D, E.** Cell migration and invasion were determined in the indicated stable cell lines knockdown of TEL2 as described in “Methods”. *n* = 3. The bars indicate the SD. *P < 0.05 and **P < 0.001 using Student's *t*-test. The representative images (100×) for the indicated stable cell lines were shown as the left panels. **F, G.** The liver metastasis *in vivo* of the indicted stable cell lines in nude mice was determined as described in “Methods”. The panel F illustrates the statistical results. *n* = 6. The bars indicate the SD. **P < 0.01 and ***P < 0.0001 using Student's *t*-test. The panel G presents the six livers having the metastatic nodules from each group, scale bars, 1 cm.

To determine the roles of TEL2 in NPC, using both S18 and 5–8F cells stably expressing ectopic TEL2 (Figure 2A), the cell proliferation was not altered by ectopic expression of TEL2 (Figures 2B and 2C). However, ectopic expression of TEL2 reduced cell migration and invasion (Figures 2D and 2E). In contrast, four different shRNAs specifically targeting different TEL2 coding regions were used. In particular, sh#1 and sh#3 were found to be highly efficient at silencing TEL2 (Supplementary Figure S2). Using the stable TEL2 shRNAs in both S26 and 6–10B cell lines (Figure 3A), the cell proliferation was not altered by knockdown of TEL2 (Figures 3B and 3C). However, both migratory and invasive abilities were significantly enhanced when TEL2 was knocked down in these cells *in vitro*, as shown in Figures 3D and 3E. Next, we carried out the hepatic metastasis model of nude mice *in vivo* [9] using S26 and its derived cells (Figure 3A). As expected, the knockdown of TEL2 resulted in a significant increase in liver metastases in S26 cells (Figures 3F and 3G). Taken together, TEL2 may negatively regulate metastasis, but not proliferation, of NPC.

### The effects of down-regulating TEL2 on NPC metastasis depend on the up-regulation of SERPINE1

**Figure 4:**
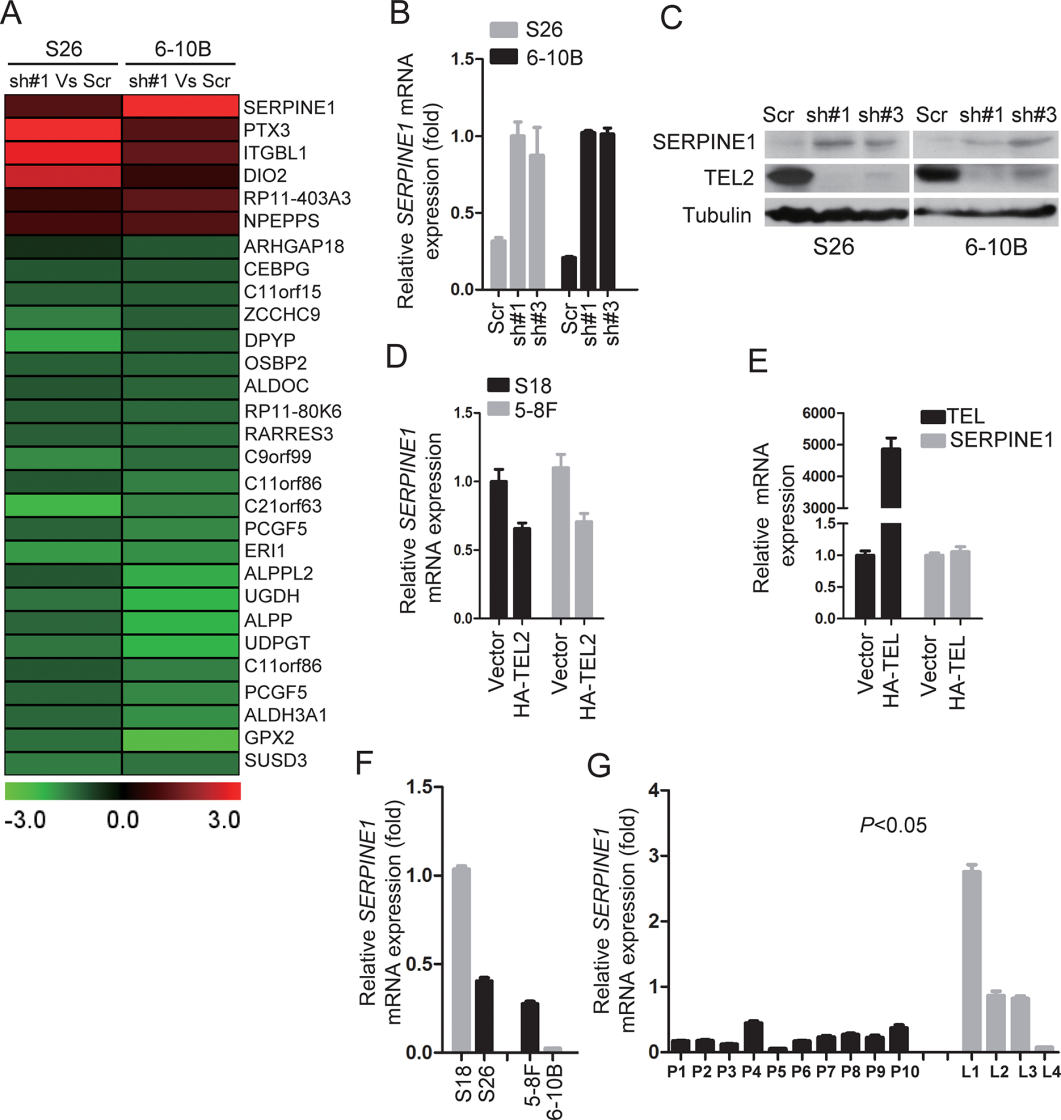
Knockdown of TEL2 increased SERPINE1 level **A.** Gene expression profiles using the stable cell line knockdown of TEL2 in S26 and 6–10B cells. **B.** The relative mRNA levels of SERPINE1 normalized to GAPDH level in the indicated cell lines, as determined by qRT-PCR (mean + SEM of triplicate samples are shown). **C.** The proteins in the indicated cell lines were analyzed by Western blotting. **D.** The relative mRNA levels of SERPINE1 in the indicated stable cell lines overexpressing TEL2 were determined as presented in B (mean + SEM of triplicate samples are shown). **E.** The relative mRNA levels of TEL and SERPINE1 in S18 cells transiently transfected with TEL or vector as determined (mean + SEM of triplicate samples are shown). **F.** The relative mRNA levels of SERPINE1 in the indicated cell lines were determined as presented in B (mean + SEM of triplicate samples are shown). **G.** The mRNA levels of SERPINE1 in the indicated tissues were measured as described in 1D. The bars indicate the SD. *P < 0.05 using Student's *t*-test.

To explore the downstream targets of TEL2 that inhibit NPC metastasis, we performed the whole genomic expression profiles using the stable TEL2 shRNAs in both S26 and 6–10B cell lines (Figure 4A). Given that TEL2 suppresses cancer metastasis, particular attention was paid to the TEL2-regulated genes implicated in these processes from this microarray analysis. SERPINE1, also named PAI-1, caught our attention, as SERPINE1 plays crucial roles in invasion and metastasis of various tumors [20, 21], and the increase of SERPINE1 by knockdown of TEL2 was validated by qRT-PCR and western blot at the mRNA and protein levels, respectively (Figures 4B and 4C). Conversely, the SERPINE1 expression was decreased in both S18 and 5–8F cells stably overexpressing HA-TEL2 (Figure 4D). However, overexression of TEL, another member in the same subfamily of TEL2, didn't change the expression of SERPINE1 (Figure 4E). As expected, SERPINE1 expression level was higher in S18 and 5–8F compared with S26 and 6–10B, respectively, as shown in Figure 4F. SERPINE1 was consistently lower in clinical NPC samples in the primary tissue compared with the metastatic LN tissues at the mRNA level, as quantified by qRT-PCR (Figure 4G). This was further supported by the fact that *LRP1*, the SERPINE1 receptor [22], was also lower in the primary NPC tissue compared with the metastatic LN tissue in the same clinical NPC samples (Supplementary Figure S3).

### TEL2 directly binds to and suppresses the SERPINE1 promoter

**Figure 5:**
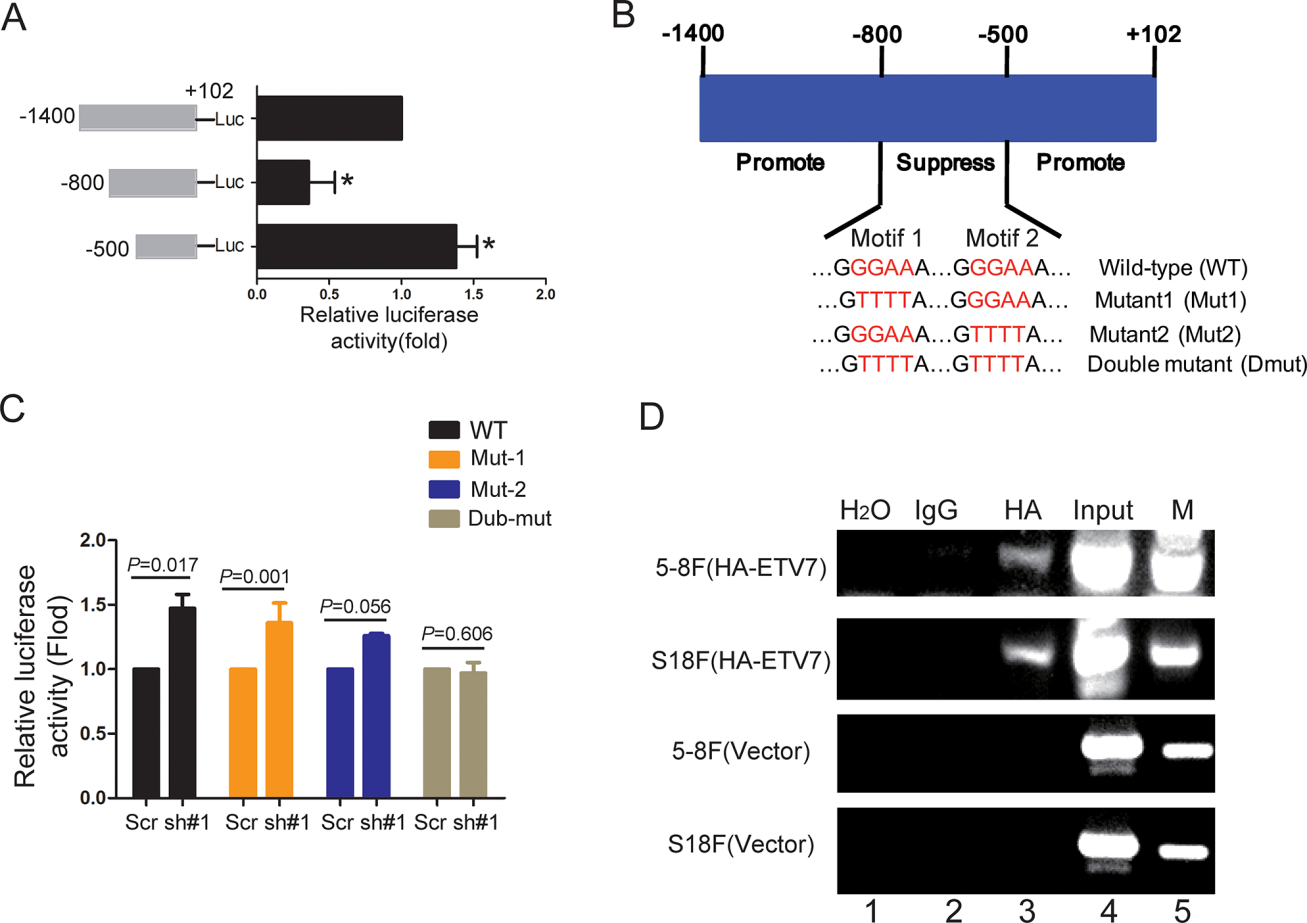
TEL2 directly binds to and suppresses the SERPINE1 promoter activity **A.** The luciferase activity of three regions of the SERPINE1 promoter in S26 cells was determined as specified in the Methods section. **B.** The schematic illustration of the suppression region within wild type SERPINE1 promoter and its mutants at both motif 1 and motif 2. **C.** The luciferase activity of wild type SERPINE1 promoter and its mutants in S26 cells after expressing TEL2 shRNA or scrambled shRNA. **D.** The indicated cells stably transfected with vector or HA-TEL2 as indicated were analyzed by ChIP assays using anti-HA as described in the Methods section.

Since TEL2 is a transcription factor, we surmised that the SERPINE1 promoter would be regulated by TEL2. To this end, three different promoter regions of SERPINE1, 100 bp to − 1400 bp, 100 bp to − 800 bp and 100 bp to − 500 bp, were cloned into the luciferase vector. As shown in Figure 5A, using the luciferase assay, the region of − 500 bp to − 800 bp was determined to be the transcriptional repression region for the SERPINE1 promoter. Strikingly, this repression region contains two TEL2 binding motifs GGAA, named as motif-1 and motif-2, as elucidated in Figure 5B. The luciferase activity was constant when both GGAA motifs were mutated into TTTT, but was still enhanced in any single motif mutant (Figure 5C), suggesting that the two motifs were important for the transcriptional suppression of SERPINE1 by TEL2. Indeed, using the ChIP assay, as shown in Figure 5D, TEL2 was detected in the two motif regions in both S18 and 5–8F cells stably overexpressing HA-TEL2, which decreased the SERPINE1 expression (Figure 4D). These results indicate that TEL2 directly binds to the SERPINE1 promoter and suppresses the transcription of SERPINE1.

### TEL-2 inhibits NPC metastasis depends on down-regulation of SERPINE1

**Figure 6:**
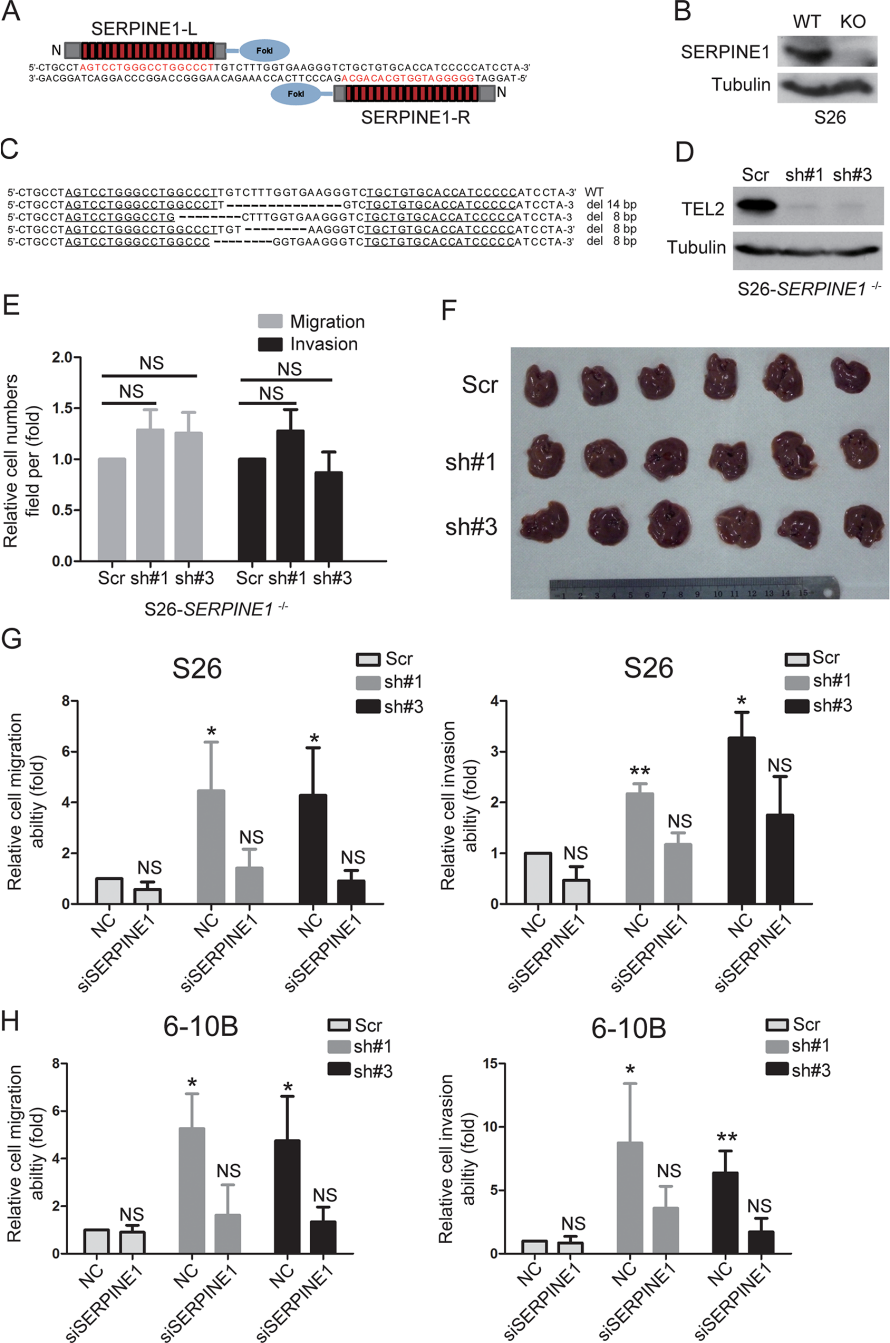
The promotion of NPC metastasis induced by down-regulating TEL2 depends on the up-regulation of SERPINE1 **A.** Schematic illustration for the layout of TALEN target sites of SERPINE1. The left and right TALENs recognize the top and bottom strands of the target sites, respectively. **B, C.** the SERPINE1 protein and DNA deleted and disrupted in S26 cells was confirmed by Western blot (B) and DNA sequence (C), respectively. **D.** S26-SERPINE1 ^−/−^ cells were transfected with lentiviruses stably expressing scrambled shRNA or shRNAs targeting TEL2 at different locis (#1 and #3), and the cells were analyzed by western blotting. **E.** The cell migration and invasion of the S26-SERPINE1^−/−^ cells were determined as in 3(D) *n* = 3. The bars indicate SD. NS: non-statistical significance using Student's *t*-test. **F.** The liver metastasis *in vivo* of the S26-SERPINE1^−/−^ cells expressing TEL2 shRNA or scrambled shRNA in nude mice was determined as in 3(F, G). The six livers without metastatic nodules from each group are shown in the image. Scale bars, 1 cm. **G, H.** The cell migration and invasion were determined as in E. *n* = 3. The bars indicate the SE. *P < 0.05,**P < 0.001. NS: non-statistical significance using Student's *t*-test.

Next, we sought to demonstrate whether down-regulation of TEL2 promoted NPC metastasis by up-regulating SERPINE1. First, the SERPINE1 knockout cell line in S26 (S26-SERPINE1^−/−^) was created through TALEN technology (Figures 6A–6C), and ectopic of SERPINE1 can rescue the abilities of migration and invasion in S26-SERPINE1^−/−^ cells (Supplementary Figure S4), indicating that SERPINE1 is really crucial for cell migration and invasion in NPC. Second, the knockdown of TEL2 was subsequently performed in the S26-SERPINE1^−/−^ cells (Figure 6D), as shown in Figures 6E and 6F, the enhancement of cell migration and invasion *in vitro* and metastasis *in vivo* by the knockdown of TEL2 was abolished in S26-SERPINE1^−/−^ cells. Third, as shown in Supplementary Figure S5, the traditional method of siRNA knockdown of SERPINE1 in the stable TEL2 shRNAs of both S26 and 6–10B cell lines was performed (Figure 3A). As expected, the migratory and invasive abilities were almost rescued to the original levels in the cells with knockdown of both SERPINE1 and TEL2 (Figures 6G and 6H, Supplementary Figure S6). Collectively, these results strongly argue that the promotion of NPC metastasis by down-regulating TEL2 depends on up-regulation of SERPINE1.

### The clinical significance of the TEL2 / SERPINE1 axis in patients with NPC

**Figure 7:**
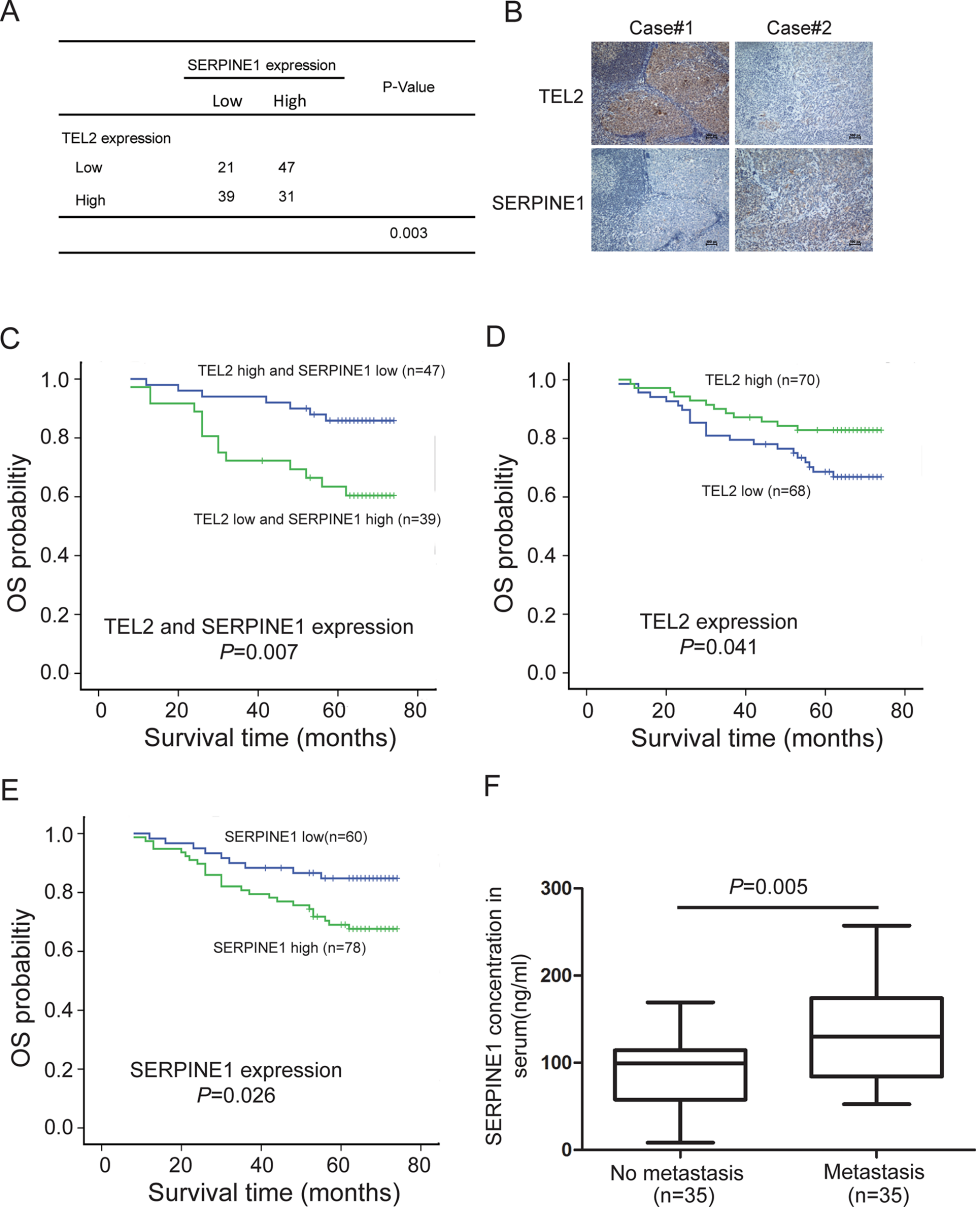
The clinical significance of the TEL2 / SERPINE1 axis in patients with NPC **A, B.** IHC for TEL2 and SERPINE1 was performed using 138 clinical NPC samples as described in the Methods section, and an inverse correlation was observed between TEL2 and SERPINE1 in NPC samples in the clinic(*P* = 0.003, χ2 tests). Scale bars in B, 100 px **C.** The subgroup with TEL2 down-expression and SERPINE1 overexpression had lower overall survival compared to that with TEL2 overexpression and SERPINE1 down-expression. **D, E.** The subgroup with high SERPINE1 (D) or low TEL2 expression (E) had poor overall survival rate compared to those with low SERPINE1 (D) or high TEL2 expression (E). **F.** The concentration of SERPINE1 in serum of NPC patients with (35 samples) or without metastasis (35 samples) as indicated was measured by ELISA. The dots represent the scores, while the bars indicate the SD.

To evaluate whether the TEL2 / SERPINE1 axis revealed in the cell culture and animal model was clinically relevant, we performed immunohistochmeistry staining for TEL2 and SERPINE1 in the 138 clinical NPC samples (Supplementary Table 2), and analyzed the correlations between the expression of TEL2 and/or SERPINE1 with clinicopathologic characteristics. As shown in Figures 7A and 7B, there was an inverse correlation between TEL2 and SERPINE1 in the primary NPC tissues. More importantly, the combination of high TEL2 expression and low SERPINE1 expression was more significantly correlated with overall survival (Figure 7C, Supplementary Table 3), although either TEL2 or SERPINE1 was found to be valuable for the prognosis of outcome in the patients with NPC (Figures 7D and 7E, Supplementary Tables 4 and 5). Because the SERPINE1 level in the plasma in patients with renal cell carcinoma was significantly higher in the group with metastasis than that without metastasis [23], we were curious to test whether this was also the case for NPC patients. As shown in Figure 7F, the SERPINE1 level in serum is higher in the NPC patients with metastasis than those without metastasis, indicating that the SERPINE1 level in the serum may be a good marker for NPC metastasis. Taken together, the TEL2 / SERPINE1 axis is clinically relevant in the patients with NPC.

## DISCUSSION

In this report, we have demonstrated for the first time that TEL2 is a key player in NPC metastasis by directly down-regulating SERPINE1. This novel axis of TEL2 / SERPINE1 may be useful to develop new strategies for treating the patients with NPC metastasis.

Transcription factors play key roles in regulating cancer metastasis, and each individual transcription factor may have different or even opposite roles among different cancer types [24, 25]. Thus, we determined that it was necessary to possess a tool to easily decipher the role of any transcription factor in metastasis for a certain cancer type. For this purpose, a customized gene microarray, including all of the human transcription factors and the known markers for EMT was generated. Using this microarray, several novel transcription factors, such as TEL2 described herein, as well as the well known markers for EMT and/or metastasis, such as Snail, Twist, Zeb1, have been showed to be significantly altered in the NPC cell lines with different metastatic capacities. Furthermore, most of these results were also validated using the NPC primary tissues and its metastatic LN tumors. In fact, TEL2 is down-regulated in the metastatic cells and tissues of NPC (Figure 1), and knockdown of TEL2 promotes migration, invasion and metastasis (Figure 3). These are in accordance with results that the patients with NPC with high levels of TEL2 have a better survival rate (Figure 7D). Therefore, we believe that our customized gene microarray is a good tool for screening new transcription factors in a variety of biological processes if appropriate cell lines and tissues are utilized.

SERPINE1, a secreted protein, promotes cancer invasion and metastasis via direct interactions with integrins, LRP1, uPA-uPAR with the ECM [23, 26], and also correlates with a poor prognosis in most cancer types [20, 26–28]. For instance, as a potential marker for the diagnosis of NPC, the SERPINE1 level in serum is significantly higher in the NPC patients compared with normal controls [28]. In this report, we showed that the higher level of SERPINE1 is an indicator of poor survival for the NPC patients and is detected in the highly metastatic cells compared with the poorly metastatic cells (Figure 4, Figure 7E). Furthermore, we found that the SERPINE1 level in the serum is higher in the NPC patients with metastasis than those without metastasis, indicating that the SERPINE1 level in the serum may be a good marker for NPC metastasis. More importantly, the promotion of NPC metastasis induced by down-regulating TEL2 depends on the up-regulation of SERPINE1 (Figure 6), and an inverse correlation was observed between SERPINE1 and TEL2 using the NPC tissues in clinic (Figure 7A). Interestingly, as determined in this report, SERPINE1 is a direct downstream target of TEL2 because TEL2 can suppress SERPINE1 by directly binding to its promoter in NPC cells (Figure 5). In addition, histone deacetylases SIRT1 and HDAC11 were reported to suppress SERPINE1 expression by binding to the SERPINE1 promoter region [29, 30]. It is possible that the effects of TEL2 on SERPINE1 may also be related to SIRT-1, HDAC11 or other histone deacetylases, which deserves to be further investigated. However, the literature has showed that SERPINE1 is promoted, not inhibited, by the ETS family members, such as ELK1, Ets-1 and ELK3, in other cancer types [31–33]. These results reinforce the notion mentioned above that each individual transcription factor may have different or even conflicting roles among different cancer types [24, 25]. Other genes such as PTX3, ITGBL1, DIO2, RP11–403A3 and NPEPPS have also been up-regulated after knockdown of TEL2. However, we currently do not know whether they are also the direct targets of TEL2, which is desired to be investigated in the future.

In summary, in NPC cells and tissues, the down-regulation of TEL2 will release its inhibition on the SERPINE1 promoter and consequently increase SERPINE1, which promotes metastasis most likely through its receptor, LRP1. This novel pathway may be valuable to design new treatments for the patients with NPC metastasis.

## MATERIALS AND METHODS

### Cell lines

Four human nasopharyngeal carcinoma cell lines (S26, S18, 6–10B, 5–8F) were cultured as described previously [8], were cultured in Dulbecco's modified Eagle's medium (DMEM, Invitrogen) supplemented with 10% fetal bovine serum (HyClone). SW480 and SW620 cells were purchased from the American Type Culture Collection (ATCC), and cultured according to the instructions. All cell lines used in this study were authenticated using short-tandem repeat profiling less than 6 months ago when this project was initiated, and the cells have not been in culture for more than 2 months.

### Plasmids

The full-length cDNAs of human TEL2 and TEL were cloned into pbabe-puro vector. HA-TEL2 was constructed into pbabe-puro vector by inserting HA tag into the TEL2 N-terminal. The promoter region of SERPINE1 was cloned into pGL3.0-basic vector. Mutations were introduced using the Quick-Change Site-Directed Mutagenesis Kit (Stratagene), and all mutations were verified by DNA sequencing.

### Antibodies

Human anti-TEL2 antibody was obtained from Sigma (HPA029033). Human anti-SERPINE1 antibody was obtained from Santa (sc-5297). Other primary antibodies used for western blotting, such as anti-E-cadherin, N-cadherin, Snail and anti-Vimentin, were obtained from BD Company. Anti-HA was obtained from Cell Signaling Technology.

### Generation of the customized gene microarray

The gene microarray of 1335 genes, including all of the human transcription factors and known markers for EMT, was generated by Bo Ao Company (Beijing, China) with 5 spots for each gene.

### RNA interference and stable lines

The effective siRNA oligonucleotides targeting SERPINE1, 5′-GGACAGACC CUUCCUCUTT-3′, were synthesized by GenePharma, Shanghai. Approximately 2 × 10^5^ S26 and 6–10B cells per well were seeded in a 60 mm culture dish on the day before transfection. Transfection of 50 nmol siRNA was performed according to the manufacturer's instructions using the LipofectamineTM RNAiMAX transfection reagent (Invitrogen). After transfection for 48 hrs, the experiment was conducted using transwell assays. The cell lines stably expressing scrambled, TEL2 short hairpin RNA (shRNA) were established by the Sigma shRNA system according to the manufacturer's instructions. The oligonucleotides for human TEL2 shRNA#1 and #3 are 5′-GCCAGATGTGAAGCTCAAATTA-3′ and 5′- GCTGCTCCTTGAT ACCCGATAT-3′, respectively.

### RNA extraction and qRT-PCR

These procedures were performed as previously described [34, 35]. Briefly, total RNA was isolated using Trizol reagent (Invitrogen) according to the manufacturer's instructions. First-strand cDNA was synthesized using Revert AidTM First Strand cDNA Synthesis Kit (MBI Fermentas). The primers used for amplifying TEL2, Snail, SERPINE1, LRP1 and GAPDH were as follows:

TEL2-F: 5′-GGGCTTACCAGCAACTTCG-3′; TEL2-R: 5′-TCTTGGCGTCCTTGTCTTCC-3′; SERPI NE1-F: 5′-AGTGGACTTTTCAGAGGTGGA-3′; SERPI NE1-R: 5′-GCCGTTGAAGTAGAGGGCATT-3′; LRP1-F: 5′-AACGAGCATAACTGCCTGGG-3′; LRP1-R: 5′-CGTA CACTGAGCACTCATCAAA-3′; TEL-F: 5′-ATCAACC TCTCTCATCGGGAA-3′ TEL-R: 5′-CAGTCTGCTATT CTCCCAATGG-3′ GAPDH-F: 5′-ACAGTCAGCCGCA TCTTCTT-3′; GAPDH-R: 5′-GACAAGCTTCCCGTTCT CAG-3′.

### Cell proliferation assay

The cell proliferation *in vitro* was assessed using CCK8 assay. Cells were seeded in 96-well plates at the density of 1,000 cells/well, and the cells were incubated for 1, 2, 3, 4, or 5 days. Ten microliters of CCK8 (Cell Counting Kit-8, Beyotime, China) was added to each well and incubated for 1.5 hours. The absorbance value (OD) of each well was measured at 450 nm. For each experimental condition, 6 wells were used.

### The transwell assays

For the transwell migration assay, 1.5 × 10^4^ (S18 and 5–8F), 3.5 × 10^4^ (S26 and 6–10B cells) in 200 μl of serum-free DMEM was added to the cell culture inserts with an 8-μm microporous filter without extracellular matrix coated (Becton Dickinson Labware, Bedford, MA). DMEM medium containing 10% FBS was added to the bottom chamber. After 24 h of incubation, the cells in the lower surface of the filter were fixed, stained, and examined using a microscope. The number of migrated cells in three random optical fields (×100 magnifications) from triplicate filters was averaged. For the *in vitro* invasion assay, the inserts of the chambers to which the cells were seeded were coated with Matrigel (Becton Dickinson Labware, Bedford, MA). The number of invading cells in three random optical fields (×100 magnifications) or each filter from triplicate inserts was averaged.

### Generation of SERPINE1 KO cells by TALEN

TALEN genomic binding sites were chosen to be 19 bp in length, and the distance of these two binding sites was 16 bp. Each binding site was anchored by a preceding T base in position “0”, which has been shown to be optimal for naturally occurring TAL proteins. TAL repeats were constructed based on the PCR-based protocol by Zhang's group [36]. And the TALEN empty vector was obtained from Addgene. TALEN left arm sequence is NI NH NG HD HD NG NH NH NH HD HD NG NH NH HD HD HD NG and TALEN right arm sequence is NH NH NH NH NH NI NG NH NH NG NH HD NI HD NI NH HD NI. S26 cells were transiently transfected with plasmids encoding the indicated TALEN pairs using lipofectamine2000 (Invitrogen, Carlsbad, CA) for 48 hrs. Single-cell clones were isolated by limiting dilution of the TALEN-treated pool. The genomic DNA of each positive clone was extracted (Qiagen) and the target locus were amplified by PCR using the primers SERPINE1-TALEN-sense: 5′-CCGGGGATAAGTCAGTCTGA G-3′, anti-sense: 5′-CCAGAGCCAGCCTTTCCTTG-3′. Then, the PCR product was sequenced and multimodal clones were selected for next step sequence analysis. The genomic DNA sequence of each multimodal clone was amplified and the PCR product was cloned into pMD-18T vector. For each cell clone, the plasmid DNAs from 40 bacterial colonies were sequenced. The whole cell lysate of positive clone was collected and SERPINE1 levels were detected by western blotting.

### The chromatin immunoprecipitation (ChIP) assay

This procedure was performed as described by ChIP kit (Active&Motif, Catalog No.53040). Briefly, 15 cm plates for each cell line to be tested were seeded with cells that were allowed to grow to 70–80% confluence. To fix cells, 1/10 growth medium volume of Complete Cell Fixative Solution was added to the existing culture media for the cells. The fixation reaction was stopped by adding 1/20 media volume of Stop Solution to the existing culture media for the cells. The cells were collected by centrifugation. After centrifugation, the nuclear pellet was resuspended in ChIP Buffer. The cell lysate was subjected to sonication and then incubated with 5 μg of antibody overnight, followed by incubation with the protein G agarose beads for 3 hrs at 4°C. Bound DNA-protein complexes were eluted and cross-links were reversed after a series of washes. Purified DNA was resuspended in TE buffer for PCR. The primers for the SERPINE1 was as follows:

SERPINE1-ChIP-F: 5′- AGAGTCTGGACACGTG GGGAGTC-3′ SERPINE1-ChIP-R: 5′- CTCCATCAAAA CGTGGAAGTTTTC-3′

### The luciferase assay

This process was carried out as described previously [37]. Briefly, the cells were plated in 12-well plates at the density of 2 × 10^5^ per well, and were transfected with 0.8 μg of promoter-luciferase plasmid. To normalize transfection efficiency, the cells were also co-transfected with 8 ng of the pRL-CMV (Renilla luciferase). After transfection for 48 hrs, the luciferase activity was measured using the Dual-Luciferase Assay kit (Promega). Three independent experiments were performed, and the calculated means and standard deviations are presented.

### Western blotting

These procedures were performed as described previously [34, 38]. Briefly, cells were collected and lysed by RIPA buffer (150 mM NaCl, 0.5% EDTA, 50 mM Tris, 0.5% NP40) and centrifuged for 20 min at 12000 rpm at 4°C. Fifty micrograms of harvested total protein was loaded, separated in 8% sodium dodecyl sulfate–polyacrylamide gradient gels and transferred onto PVDF membranes followed by blocking with 5% non-fat milk for 2 hours at room temperature. Membranes were incubated with primary antibody and horseradish peroxidase-conjugated secondary antibody, and then detected using the ECL chemiluminescence system (Pierce, Rockford, USA).

### Animal experiments

All animal works were performed in accordance with protocols approved by Research Animal Resource Center of Sun Yat-sen University. Male athymic mice between 5 and 6 weeks of age were obtained from Shanghai Institutes for Biological Sciences (Shanghai, China). All the animal studies were conducted in accordance with the principles and procedures outlined in the guidelines of Institutional Animal Care and Use Committee at Sun Yat-sen University Cancer Center. The spontaneous spleen-liver metastasis model has been published previously [9]. Briefly, a total of 3 × 10^5^ cells in 30 μl were injected into spleens of laparotomized mice using insulin syringes (Becton Dickinson). At 32 days after tumor cell inoculation, the experiment was terminated. The metastatic nodules in each liver were counted.

### Clinical samples

This procedure was approved by the Institutional Review Board of Sun Yat-sen University and written informed consent was obtained from the patients prior to sample collection. A total of 168 NPC samples were obtained. The ages of the patients ranged from 6 to 52 years old. Tissue blocks prepared from NPC tissues and LN metastases were sectioned for performing immunohistochemistry (IHC) for TEL2 and SERPINE1. Clinicopathologic features of these 138 patients are shown in Supplementary Table 2. For ELISA assays, 70 serum samples from the patients with NPC were stored at Sun Yat-sen University Cancer Center. In addition, we also collected 18 pairs of the clinical samples of colon cancers at Sun Yat-sen University Cancer Center.

### ELISA

An ELISA (enzyme-linked immune sorbent assay) was performed using the ELISA SERPINE1 kit according to the manufacturer's instructions (eBioscience, #BMS2033). The serum samples were diluted 1:50 in phosphate buffered saline prior to detection.

### IHC and histological evaluation

This procedure was described previously [8, 34]. Immunohistochemical analysis was performed on 3 μm sections. The primary antibodies against TEL2 and SERPINE1 were diluted 1:250 and 1:100, respectively, and were incubated at 4°C overnight in a humidified container. After washing with PBS three times, the tissue slides were treated with a non-biotin horseradish peroxidase detection system according to manufacturer's instructions (Dako). The IHC staining was evaluated by two independent pathologists majoring in NPC (Drs. Mei Li and Rongzhen Luo). The protein expressions of TEL2 and SERPINE1 were evaluated by using the semiquantitative IRS (immunoreactive score) scale according to Remmele and Stegner [8, 39]. Generally, the SERPINE1 signal was detected in the cytoplasm, membranes and extracellular matrix, whereas the TEL2 signal was detected in the cytoplasm and the nucleus. The staining intensity for either TEL2 or SERPINE1 has four classes in NPC tissues, namely 0, 1, 2 and 3 and was designated as absent, weak, moderate and strong signals, respectively. The percentage of stained cells was categorized as 0, 1, 2, 3 and 4 to indicate no staining, 1–10%, 11–50%, 51–80% and 81–100% of stained cells, respectively. The score for each tissue was calculated by multiplying the staining value by the percentage category value, and the average of the scores from the two pathologists was used as the final score. For histological evaluation, mouse liver metastatic nodules were resected and fixed in 4% paraformaldehyde, followed by routine histology evaluation.

### Study approval

Animal experiments were approved by the Animal Research Committee of Sun Yat-sen University Cancer Center and were performed in accordance with established guidelines. The use of human nasopharyngeal carcinoma and colon cancer tissues was reviewed and approved by the ethical committee of Sun Yat-sen University Cancer Center, and the informed consent has been obtained. The samples were retrospectively acquired from the surgical pathology archives of Sun Yat-sen University Cancer Center.

### Statistics

Data are presented as the mean ± SD. Student's *t*-test or Mann-Whitney U test was employed to compare the values between subgroups. The association between TEL2 with SEPRINE1 abundance was assessed using χ2 tests. The cutoff values of TEL2 and SEPRINE1 were 8 and 4, respectively, according to their receiver operating characteristic (ROC) curves. The patients were then divided into high and low expression groups. Survival curves were constructed using the Kaplan-Meier method and compared using the log-rank test. Statistical analyses were performed using the SPSS 16.0 software (Chicago, IL). *P* < 0.05 was considered significant, *P* < 0.001 was considered strongly significant.

## SUPPLEMENTARY FIGURES AND TABLES


