# Supplementary material for: TEL2 suppresses metastasis by down-regulating SERPINE1 in nasopharyngeal carcinoma
Source: Oncotarget. 2015 Aug 13;6(30):29240–53. doi: 10.18632/oncotarget.5074 (PMC4745723; doi:10.18632/oncotarget.5074)
Supplement: Supplementary file 1 [file oncotarget-06-29240-s001.pdf]

SUPPLEMENTARY TABLES AND FIGURES

Supplementary Table S1: The results of gene microarray

	Fold change	
Gene name	LN Vs NPC	S18 VS S26
N-cadherin	5.38	2.89
Vimentin	2.58	9.91
TGFB1	1.34	8.27
Snail	1.11	4.08
ZEB1	4.305	4.08
E-cadherin	0.26	0.17
FHL1	27.9	7.41
HDAC4	2.59	2.56
ETV5	2.95	3.63
HLX1	2.46	3.16
TEAD1	2.43	3.16
ZNF319	1.7	2.72
KLF5	0.08	0.4
EVI1	0.4	0.317
ZNF339	0.27	0.05
HES2	0.43	0.23
FOXA1	0.12	0.39
TEL2	0.49	0.25

Supplementary Table S2: Clinical Characteristics Of 138 NPC Patients

Characteristics	No.(%)
**Age, years**	
Median	47
Range	19–73
**Gender**	
Male	102(73.9)
Female	36(26.1)
**T stage**	
T1	11(8.0)
T2	38(27.5)
T3	58(40.2)
T4	31(22.5)
**N stage**	
N0	21(15.2)
N1	65(47.1)
N2	41(29.7)
N3	11(8.0)
**M stage**	
M0	132(95.7)
M1	6(4.3)
**Clinical staging**	
I	4(2.9)
II	31(22.5)
III	57(41.3)
IV	46(33.3)
**Death**	
Yes	34
No	104

Supplementary Table S3: The correlation between the combination of TEL2 with SERPINE1 level and clinicopathologic characteristics of patients with NPC

	TEL2 and SERPINE1 expression				TEL2 and SERPINE1 expression			
Characteristics	*n*	TEL2 Low SERPINE1 High	TEL2 High SERPINE1 Low	Chi-square test	*n*	TEL2 Low SERPINE1 Low	TEL2 High SERPINE1 High	Chi-square test
		No. cases	No. cases	*p* value		No. cases	No. cases	*p* value
**Gender**				0.709				0.978
Male	60	32	28		42	17	25	
Female	26	15	11		10	4	6	
**Age**				0.797				0.330
≤47	52	29	23		23	11	12	
>47	34	18	16		29	10	19	
**Clinical Stage**				0.457				0.135
I-II	21	10	11		14	8	6	
-IV	65	37	28		38	13	25	
**T classification**				0.783				0.436
T1–2	30	17	13		19	9	10	
T3–4	56	30	26		33	12	21	
**N classification**				0.058				0.084
N0–1	50	23	27		35	17	18	
N2–3	36	24	12		17	4	13	
**Death**				**0.005**				0.870
Yes	21	17	4		13	5	8	
No	65	30	35		39	16	23	

Supplementary Table S4: The correlation between TEL2 expression level and clinicopathologic characteristics of patients with NPC

		TEL2 expression		
**Characteristics**	***n***	**Low or none**	**High**	**Chi-square test**
		**No. cases**	**No. cases**	***p* value**
**Gender**				0.919
Male	102	50	52	
Female	36	18	18	
**Age**				0.298
≤47	75	40	35	
>47	63	28	35	
**Clinical Stage**				0.493
I-II	35	19	16	
III-IV	103	49	54	
**T classification**				0.509
T1–2	49	26	23	
T3–4	89	42	47	
**N classification**				0.629
N0–1	86	41	45	
N2–3	52	27	25	
**Death**				**0.038**
Yes	34	22	12	
No	104	46	58	

Supplementary Table S5: The correlation between SERPINE1 expression level and clinicopathologic characteristics of patients with NPC

		SERPINE1 expression		
**Characteristics**	***n***	**Low or none**	**High**	**Chi-square test**
		**No. cases**	**No. cases**	***p* value**
**Gender**				0.799
Male	102	45	57	
Female	36	15	21	
**Age**				0.631
≤47	75	34	41	
>47	63	26	37	
**Clinical Stage**				0.059
I-II	35	20	15	
III-IV	103	40	63	
**T classification**				0.803
T1–2	49	22	27	
T3–4	89	38	51	
**N classification**				0.007
N0–1	86	45	41	
N2–3	52	15	37	
**Death**				**0.021**
Yes	34	9	25	
No	104	51	53	

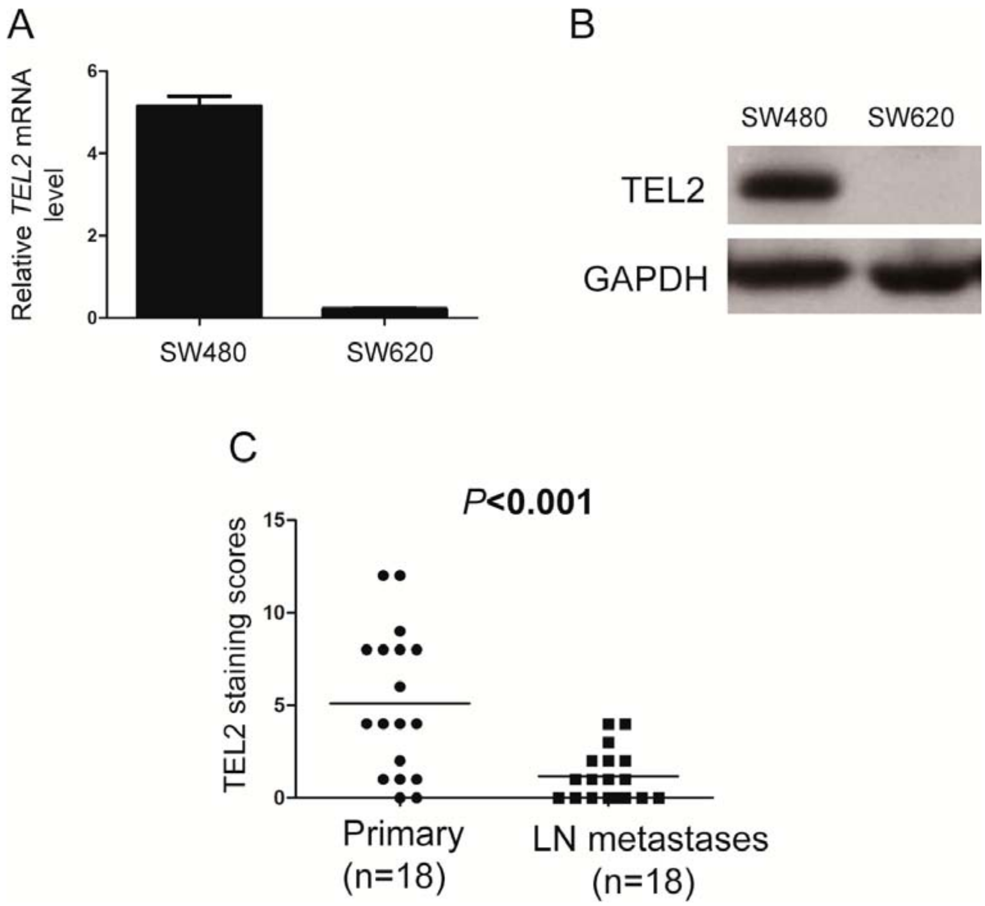

Supplementary Figure S1: TEL2 is down-regulated in metastatic cancer cell lines and tissues. A. The relative mRNA levels of *TEL2* normalized to GAPDH level in the indicated cell lines determined by qRT-PCR (mean + SEM of triplicate samples are shown). **B.** The proteins in the indicated cell lines were analyzed by Western blotting. **C.** The statistical results of IHC for the colon cancer primary tissues and their paired LN metastases using a paired *t* test ($P < 0.001$). The dots represent the scores, while the bars indicate the SD. $n = 18$. Primary: primary colon cancer tissues, LN metastases: the metastatic tumor tissues in lymph node.

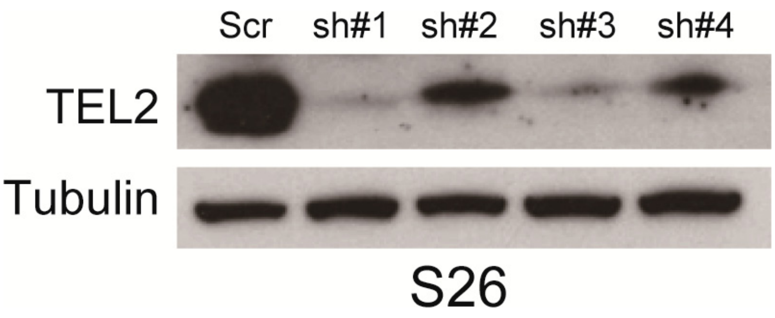

Supplementary Figure S2: sh#1 and sh#3 were highly efficient at silencing TEL2. S26 cells were stably transfected with four different shRNAs against TEL2 and subjected to Western blotting.

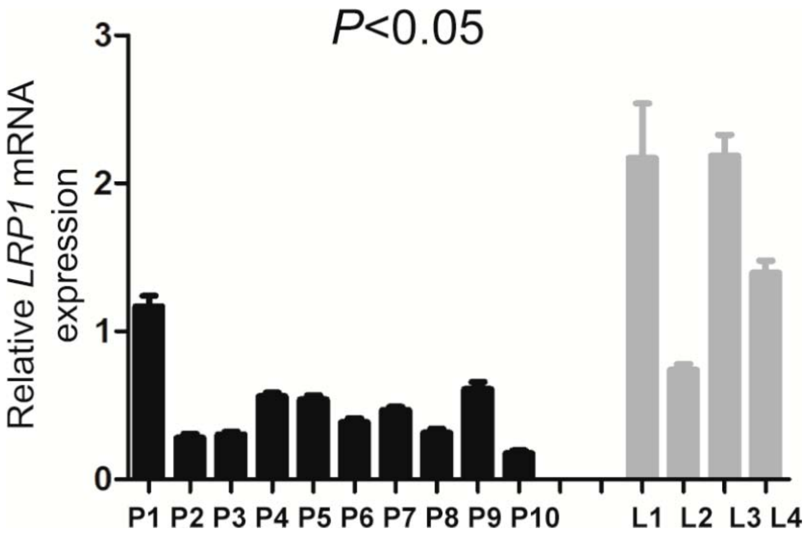

Supplementary Figure S3: The mRNA levels of *LRP1* were higher in the metastatic NPC tumor tissues in lymph node than the NPC primary tissues. The mRNA levels of *LRP1* in the indicated tissues were measured by qRT-PCR as described in Figure 4G. *P*: NPC primary tissues, $n = 10$, L: the metastatic tumor tissues in lymph node, $n = 4$. The bars indicate the SD. $*P < 0.05$ using Student's *t*-test.

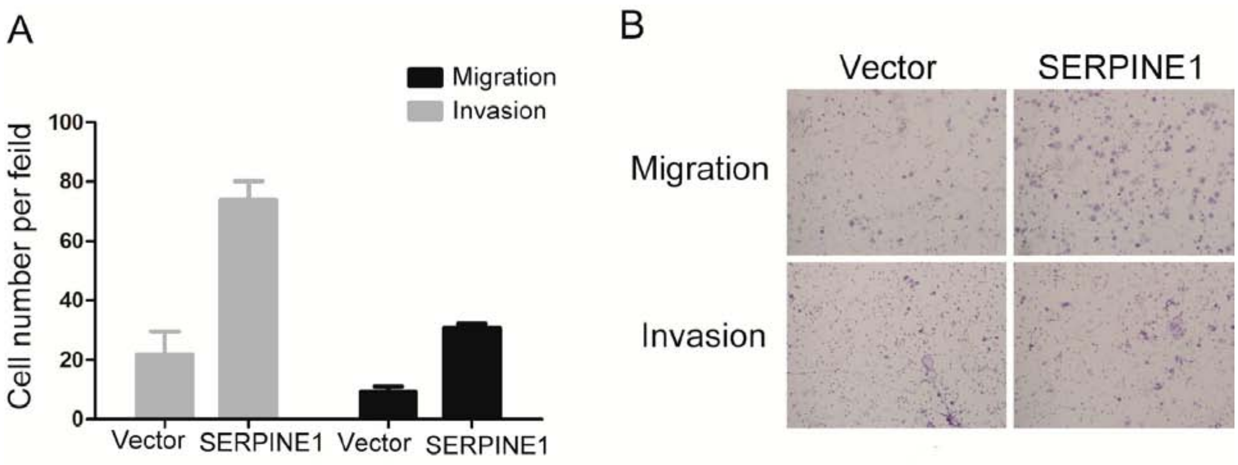

Supplementary Figure S4: Ectopic of SERPINE1 can rescue the abilities of migration and invasion in S26-SERPINE1$^{-/-}$ cells. A, B. Cell migration and invasion were determined in S26-SERPINE1$^{-/-}$ cells stably overexpressing vector or SERPINE1 as indicated. $n = 3$. The bars indicate the SD (A). The representative images (100×) for the indicated cells are shown (B).

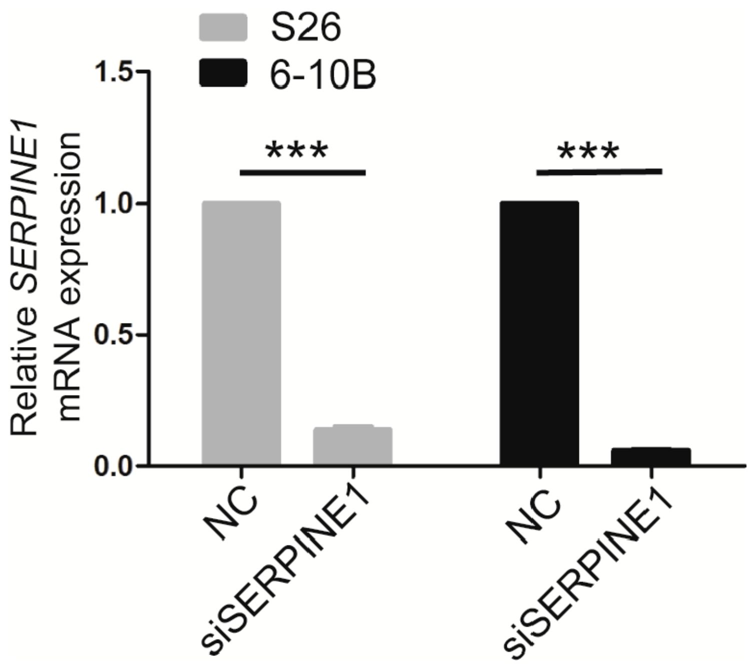

Supplementary Figure S5: The knockdown efficacy of TEL2 by siRNA in S26 and 6–10B cells. The indicated stable cells were transfected with siSERPINE1 or negative control siRNA (NC) as indicated for 48 hrs, and the SERPINE1 mRNA level was measured by qRT-PCR. $n = 3$. The bars indicate the SD. ***$P < 0.001$ using Student's *t*-test.

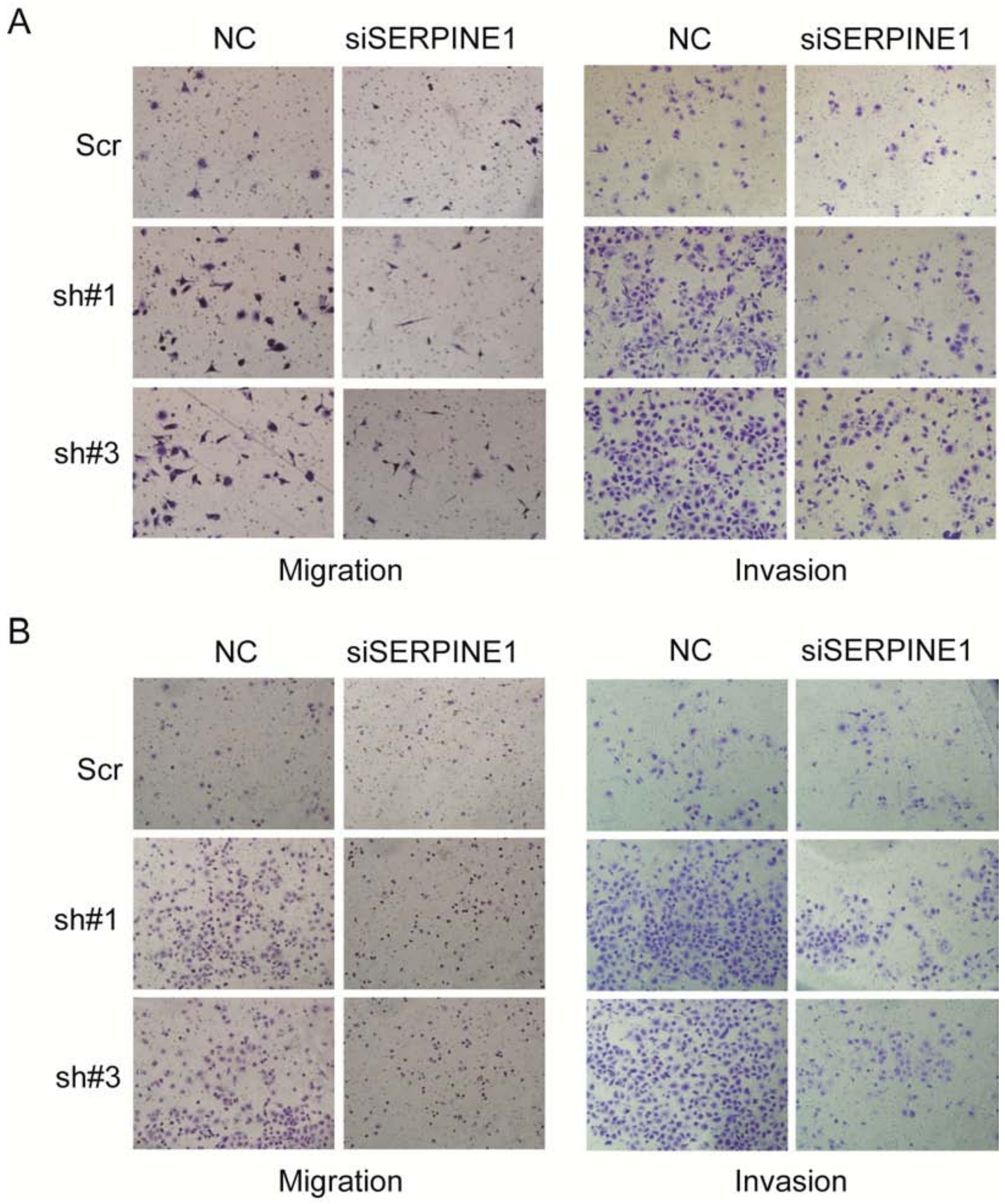

Supplementary Figure S6: The promotion of cell migration and invasion by knockdown of TEL2 was abolished by simultaneously knocking down of SERPINE1 in NPC cells. The cell migration and invasion were determined in both S26 cells **A.** and 6–10B cells **B.** under indicated conditions, as described in the Methods section. $n = 3$. The representative images (100×) for the indicated stable cell lines were shown.
